# Malaria and the incidence of COVID-19 in Africa: an ecological study

**DOI:** 10.1186/s12879-023-08032-2

**Published:** 2023-02-03

**Authors:** Farrokh Habibzadeh

**Affiliations:** Global Virus Network (GVN), Middle East Region, Shiraz, Iran

**Keywords:** COVID-19, SARS-CoV-2, Malaria, Innate immunity, Africa, Correlation of data

## Abstract

**Background:**

It has been shown that stimulation of innate immunity may provide temporary protection against a variety of infectious diseases. Malaria has been shown to induce a robust innate immune response. This study was conducted to test the hypothesis that if the cumulative number of cases diagnosed with COVID-19 per 100,000 population was correlated with the prevalence of malaria in African countries where both malaria and COVID-19 were prevalent.

**Methods:**

In this ecological study, the cumulative incidence of COVID-19 and the prevalence of malaria were compared in 53 African countries. A negative binomial regression analysis with the cumulative incidence of COVID-19 as the dependent variable, and the human development index (HDI) and the prevalence of malaria, as independent variables, were used.

**Results:**

The mean cumulative incidence of COVID-19 was 522 cases per 100,000. Each 0.1 unit increase in HDI was associated with 2.4-fold (95% confidence interval 1.8–3.1) increase in the cumulative incidence of COVID-19. Prevalence of malaria was also independently associated with the cumulative incidence; each 10% increase in the prevalence was associated with 28% (10–41%) decrease in the cumulative incidence of COVID-19.

**Conclusions:**

Malaria might protect people against SARS-CoV-2 through the stimulation of innate immunity. Stimulation of the innate immune system could be the first line of defense in the pandemic preparedness arsenal.

**Supplementary Information:**

The online version contains supplementary material available at 10.1186/s12879-023-08032-2.

## Background

Innate immunity is a part of the immune system that can provide temporary non-specific protection against a wide range of infectious agents [1, 2]. Live attenuated vaccines such as Bacillus Calmette-Guérin (BCG); measles, mumps, and rubella (MMR) vaccine; and oral polio vaccine (OPV) have been shown to stimulate the innate immune system that provides non-specific protection against unrelated infections including influenza and severe acute respiratory syndrome coronavirus 2 (SARS-CoV-2) [3–5]. It has been shown that BCG vaccination can non-specifically protect newborns against non-tuberculous infectious diseases during the neonatal period [5]. In a systematic review, meta-analysis of five clinical trials showed that BCG vaccination is associated with 30% reduction in all-cause mortality in children aged less than 5 years [6]. Analysis of 10 cohort and two case–control studies evaluating the effect of measles vaccination on all-cause mortality in children residing in seven developing countries, revealed that the mortality was decreased by 30–86%, far larger than the value expected based on the protection provided by the vaccine only against measles [3]. Controlled clinical trials conducted in 1960’s and 70’s on more than 60,000 people indicated that OPV vaccination is associated with an almost fourfold decrease in mortality and morbidity attributable to influenza [7, 8]. OPV has also been shown to confer protection against SARS-CoV-2. A recent ecological study showed that countries using OPV have a lower cumulative incidence of COVID-19 compared to those using inactive polio vaccine [9]. A cohort study also revealed that indirect exposure to the attenuated poliovirus excreted by children who had received OPV was associated with a significant decrease in the incidence of symptomatic SARS-CoV-2 infection in their mothers for more than 6 months [4]. A recent randomized clinical trial conducted on more than 1000 adults aged between 18 and 65 years, revealed that 3 months after OPV vaccination, the risk of laboratory-confirmed SARS-CoV-2 infection is decreased by 43% [10]. Also, a recent ecological study showed that the use of OPV compared with the inactivated polio vaccine, is associated with a lower mother-to-child human immunodeficiency virus transmission rate too [11]. All these non-specific protective effects are believed to be conferred through the stimulation of the innate immunity and are not just limited to live attenuated vaccines. Stimulation of the innate immunity by human rhinovirus can also block SARS-CoV-2 virus replication [12].

Malaria infection can induce a robust innate immune response [13], and if this stimulation conferred protection against SARS-CoV-2, then we expect to observe lower incidence rates of SARS-CoV-2 infection in areas where malaria is prevalent. This study was thus conducted to test if the cumulative number of cases diagnosed with COVID-19 per 100,000 population was correlated with the prevalence of malaria in African countries where both malaria and COVID-19 were prevalent.

## Methods

### Source of data

In this ecological study, the cumulative number of COVID-19 cases diagnosed was retrieved for each of 53 African countries from “Our World in Data” website on April 19, 2021 (publicly available from https://ourworldindata.org/covid-cases) [14]. The prevalence of malaria for all ages and sexes in 2019 were also retrieved for each country from the Global Burden of Disease (GBD 2019) Collaborative Network website on July 11, 2022 (publicly available from https://vizhub.healthdata.org/gbd-results/) [15].

There were other variables that might affect the incidence of COVID-19 and the prevalence of malaria, including the quality of the health care system and the surveillance infrastructures that would certainly influence the detection rate and reporting of the diseases. Therefore, an important part of this study was to account for confounding factors that might influence the conclusions.

The population and population density, the median age and the life expectancy at birth, the gross domestic product (GDP) *per capita*, and the human development index (HDI)—a measure reflecting levels of social and economic development in a country [16]—were also retrieved for each country from “Our World in Data” website on April 19, 2021 [14].

The stringency index is a composite metric calculated from nine response indicators—school closures, workplace closures, cancellation of public events, restrictions on public gatherings, closures of public transport, stay-at-home requirements, public information campaigns, restrictions on internal movements, and international travel controls [17]. It reflects the level of strictness of the government policies primarily aiming at restricting people’s behavior (mainly applied through lockdown), and ranges from 0 (no restriction) to 100 (highest levels of restrictions). The index was also retrieved for each of 53 African countries for each day before April 9, 2021, from “Our World in Data” website (publicly available from https://ourworldindata.org/grapher/covid-stringency-index) [18]. The mean stringency index for each country was used for data analyses. Data about the type of polio vaccine used by each country was provided by the World Health Organization Global Polio Eradication Initiative (GPEI). The data for generating the Africa map were retrieved from Natural Earth, a public domain map dataset (https://www.naturalearthdata.com/) [19].

### Statistical analysis

*R* software version 4.2.0 (*R* Project for Statistical Computing) was used for data analysis. Normal probability plot (using *geom_qq* and *stat_qq* of the *ggplot2* package) was used to determine whether a continuous variable follows normal distribution. Wilcoxon rank sum test (using *wilcox.test* function) was used to compare the distribution of two continuous variables not normally distributed. Continuous variables were expressed as median (interquartile range [IQR]). Spearman’s *ρ* (using *rcorr* function of *Hmisc* package) was used to determine the extent of correlation between continuous variables not normally distributed.

The cumulative incidence of COVID-19 was calculated by dividing the number of cases diagnosed in each country by its population at the midpoint of the study period multiplied by 100,000. Because the incidence (dependent variable in our analysis) had overdispersion, negative binomial regression analysis was used (with function *glm.nb* of the *R* package *MASS*). The GDP *per capita*, median age, and life expectancy at birth had a significant high correlation with HDI; thus, to avoid multicollinearity, we have only used HDI and the prevalence of malaria in each country as independent variables in the model. Outliers were included in all data analysis. A p value < 0.05 was considered statistically significant.

## Results

**Fig. 1:**
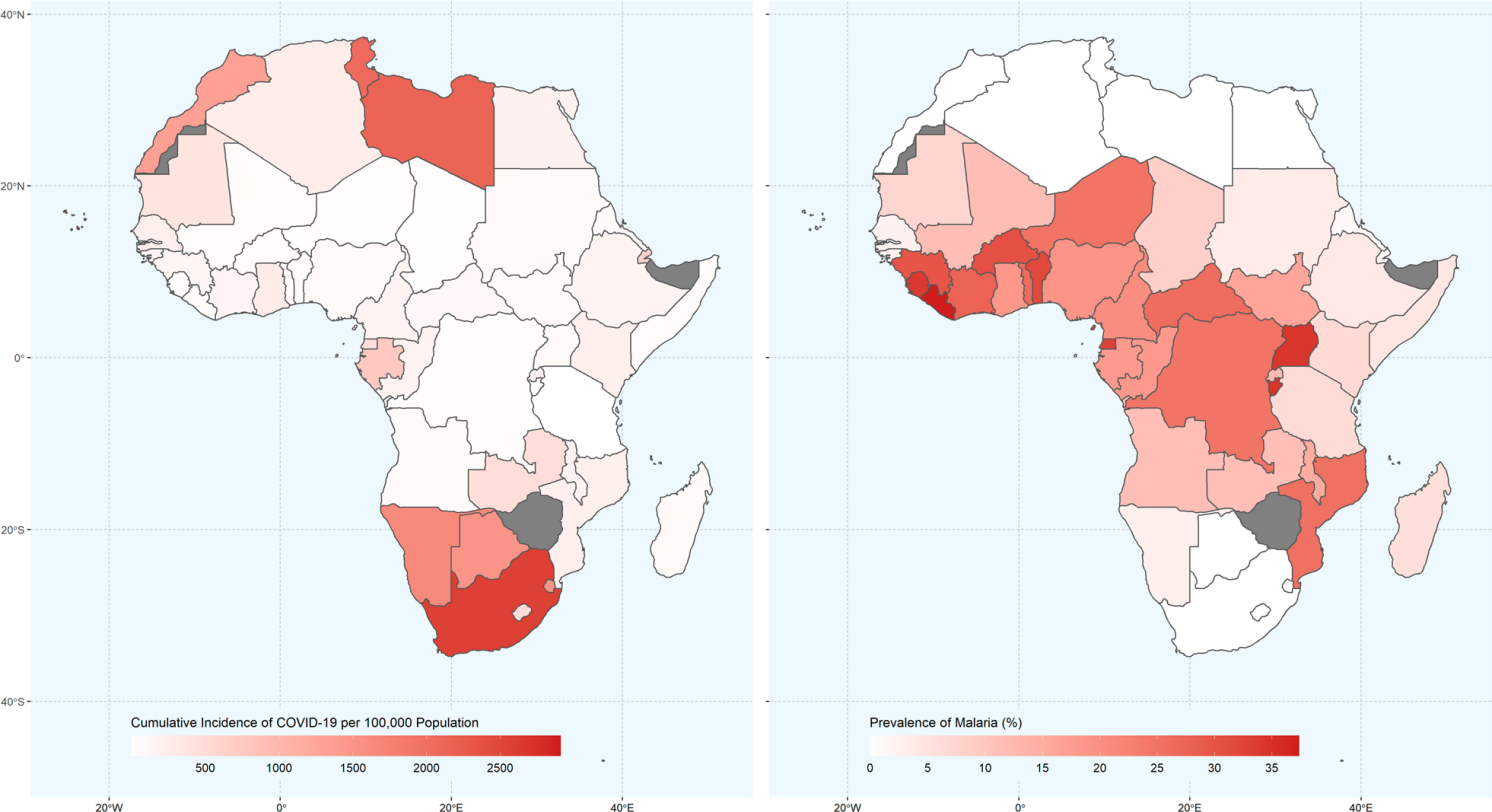
Distribution of cumulative incidence of COVID-19 (left panel) and prevalence of malaria (right panel) in African countries. Note that the number of cases diagnosed with COVID-19 per 100,000 population is lower where the malaria is prevalent and higher where the prevalence is low. Complete data were not available for gray areas. Made with Natural Earth (https://www.naturalearthdata.com/)

**Fig. 2:**
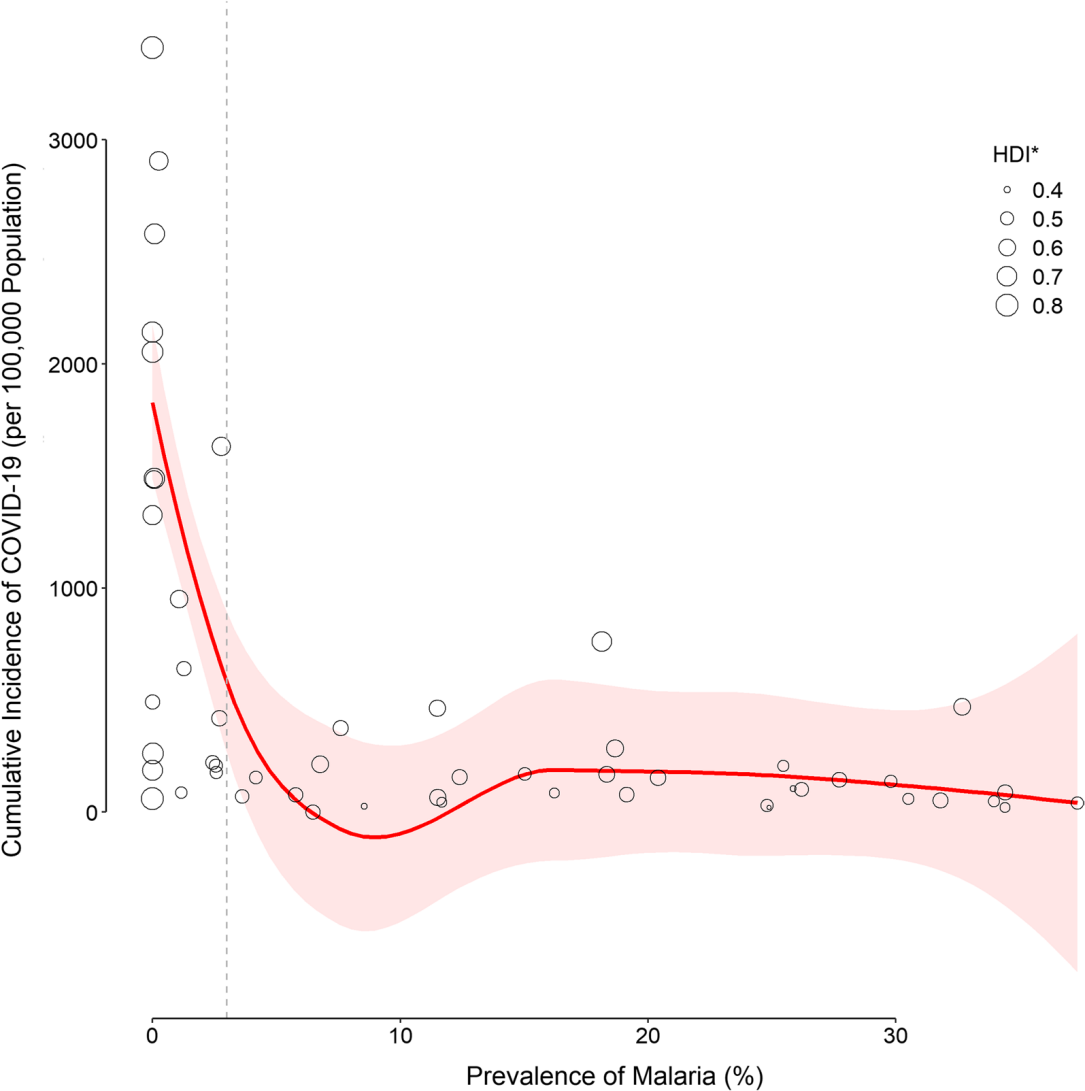
Distribution of the cumulative incidence of COVID-19 in African countries with different prevalence of malaria stratified by the human development index. The curve was smothed and drawn based on the predicted values derived from the negative binomial regression (Table 2). The shaded area represnts the 95% confidence interval of the curve. The vertical gray dashed line corresponds to an arbitrary chosen malaria prevalence of 3%. *HDI: human development index

**Fig. 3:**
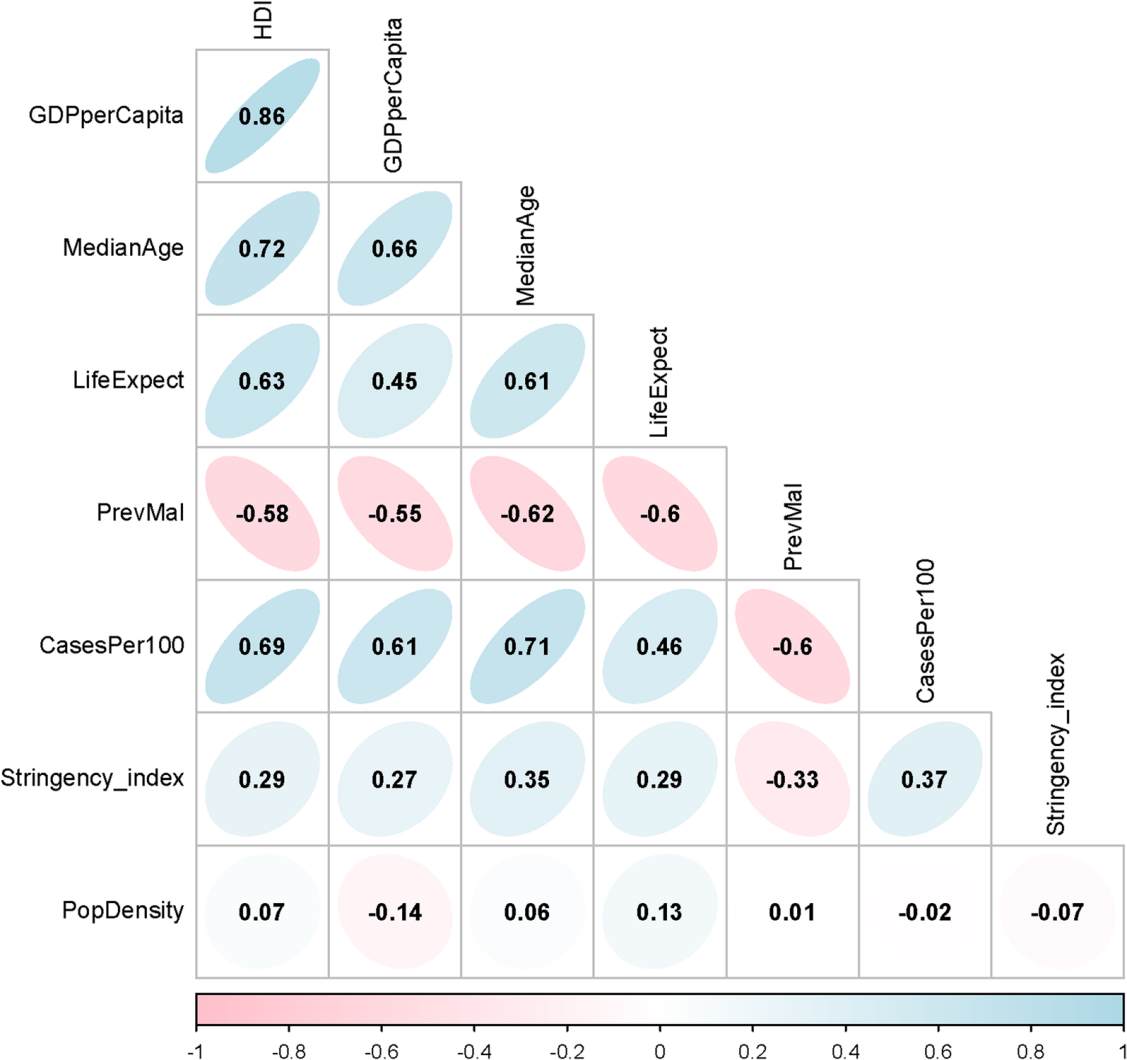
Values of Spearman’s *ρ* between each of two studied continuous variables. HDI represents human development index; GDPperCapita, gross domestic product per capita; MedianAge, median age; LifeExpect, life expectancy; PrevMal, prevalence of malaria; CasesPer100, cumulative incidence of COVID-19 per 100,000 population; and PopDensity, population density

Data for 53 African countries were studied. All the 53 studied countries used OPV. None of the studied variables followed normal distribution (Additional file 1: Fig. S1). Median and IQR of the studied variables are presented in Table 1. The distribution of COVID-19 incidence and malaria prevalence was not uniform in the studied countries (Fig. 1); they were negatively correlated (Spearman’s *ρ* = −0.6, p < 0.001)—the number of cases diagnosed with COVID-19 per 100,000 population was lower in countries where the malaria is prevalent and vice versa (Figs. 1 and 2). There were 20 countries with a malaria prevalence < 3% (an arbitrary chosen cut-off value) and 33 with the prevalence ≥ 3%. The cumulative incidence of COVID-19 and HDI in countries in the former group were significantly (p < 0.001) higher than those in countries in the latter group (Table 2, Additional file 1: Fig S2). HDI had a significant (p < 0.001) correlation with both the cumulative incidence of COVID-19 (*ρ* = 0.69) and malaria prevalence (*ρ* = −0.58). HDI ranged from 0.39 in Niger to 0.80 in Mauritius and Seychelles where the prevalence of malaria was reported to be nil. HDI was also positively correlated with GDP *per capita*, median age, and life expectancy at birth (*ρ* > 0.63, p < 0.001) (Fig. 3).

**Table 1:** Median (IQR) of studied variables in 53 African countries

Variable	Median (IQR)	Range (min to max)
CasesPer100^a^	169 (71–471)	1–3411
HDI^b^	0.54 (0.48–0.61)	0.39–0.80
Malaria prevalence (%)	7.6 (1.2–24.8)	0.0–37.3
GDP *per capita* (× 1000 US$)	2.8 (1.6–6.5)	0.7–26.4
Median age (years)	19 (18–22)	15–37
Life expectancy at birth (years)	64 (61–67)	53–77
Population density (people/km^2^)	65 (23–110)	3–623
Stringency index	48 (41–57)	14–76

^a^Cumulative incidence of COVID-19 per 100,000 Population

^b^Human development index

**Table 2:** Median (IQR) of cumulative incidence of COVID-19 per 100,000 population (CasesPer100) and human development index (HDI) in countries stratified by malaria prevalence

Variable	Countries with malaria prevalence		p value
	< 3%, (n = 20)	≥ 3%, (n = 33)	
CasesPer100	796 (218–1737)	89 (54–169)	< 0.001
HDI	0.66 (0.53–0.73)	0.52 (0.46–0.55)	< 0.001

The cumulative incidence of COVID-19, the dependent variable in our regression model, had a mean of 522 cases per 100,000; the variance exceeded 6.4 × 10^5^. For overdispersion, we used a negative binomial regression. The model could explain 90% (Nagelkerke’s *R*^2^ = 0.90) of the variance observed in the incidence (Table 3).

**Table 3:** Results of negative binomial regression

Variable	Coefficient (95% CI)	Adj IRR^a^ (95% CI)	p value
HDI^b^	8.73 (6.09–11.41)	61.96 (4.40–902.90) × 10^2^	< 0.001
Malaria prevalence (%)	−0.03 (−0.05 to −0.01)	0.97 (0.95–0.99)	0.003
Intercept	1.14 (−0.51 to 2.83)	3.13 (0.60–16.87)	0.149

Nagelkerke’s *R*^2^ = 0.90

^a^Adjusted incidence rate ratio

^b^Human development index

In negative binomial regression, if *C*_*i*_ is the coefficient of the ith independent variable in the model, then the adjusted incidence rate ratio (IRR) is exp(*C*_*i*_). For example, the adjusted IRR for HDI is 6196 [exp(8.7316), Table 3], which means that keeping other variables in the model constant, an increase of 1 unit in HDI is associated with an increase of 6196-fold in the dependent variable, the cumulative incidence of COVID-19. However, the HDI can only vary from 0 to 1, and thus, here the IRR corresponding to a smaller change (0.1 unit increase in HDI)—exp(0.1 × *C*_*i*_)— was calculated and reported. In a similar way as 1% increase in malaria prevalence was a little change, the IRR corresponding to a larger change of 10% increase in malaria prevalence—exp(10 × *C*_*i*_)—was presented here.

Based on the computations mentioned above, HDI was found to be an independent predictor of the cumulative incidence of COVID-19; each 0.1 unit increase in HDI was associated with 2.4-fold [exp(0.1 × 8.7316); 95% confidence interval (CI) 1.8–3.1] increase in the incidence (Table 3). Prevalence of malaria was also independently associated with the cumulative incidence of COVID-19; each 10% increase in the prevalence was associated with 28% (95% CI 10–41%) decrease in the cumulative incidence of COVID-19 (Table 3).

## Discussion

The mean cumulative incidence of COVID-19 in 53 African countries (522 cases per 100,000, all using OPV) was much lower than the mean value reported for all 146 countries using OPV in the world (1580 cases per 100,000) [9]. The observed relatively lower incidence of COVID-19 in Africa could be attributed to its different demographic pyramid, difference in the prevalence of pre-existing conditions, genetic build-up of the population, dissimilar sociocultural dynamics, and the trained immunity [20]. It could also be due to under-diagnosis and under-reporting of COVID-19 cases, a consequence of poor infrastructure of the health care system in some of the studied countries.

The cumulative incidence of COVID-19 was negatively correlated with the prevalence of malaria. Wherever the prevalence of malaria was high, the incidence of COVID-19 was relatively low (Figs. 1 and 2). This strong significant negative correlation still held after the model was adjusted for HDI, a confounding variable, so that each 10% increase in the prevalence of malaria was associated with 28% decrease in the incidence of COVID-19. HDI had a strong correlation with GDP *per capita*, the median age, and the life expectancy in the studied countries (Fig. 3). This is not surprising; developed by the United Nations Development Programme (UNDP), HDI is a composite idex reflecting the average achievement in key dimensions of human development [16]. It is calculated based on the life expectancy at birth (and thus, the median age of the population), gross national income (and thus, the GDP) *per capita*, and other factors. Therefore, the regression model was only adjusted for HDI (to avoid multicollinearity), and it could explain 90% of the observed variations.

Many factors might affect the cumulative incidence of COVID-19 and the prevalence of malaria. The quality of the health care system and the surveillance infrastructures would certainly influence the detection rate and reporting of cases with COVID-19 and malaria. Countries with higher HDI have expectedly a better health infrastructure and reporting system and this would explain the observed strong positive correlation between HDI and the cumulative incidence of COVID-19 and the association found between the two variables in the model. Higher HDI may be translated into a better health infrastructure and sanitation, hence, better control of malaria—the prevalence of malaria was nil in Mauritius and Seychelles where the HDI was 0.8. All the studied 53 African countries used OPV. Therefore, the type of vaccine could not be considered a factor in our analyses [9].

It has been shown that malaria can induce the innate immunity [13]. The parasite and its hemozoin induce the innate immunity through complex carefully orchestrated interactions among immunological signals, certain cell metabolites, and epigenetic reprogramming [13, 21]. Kenyan children naturally infected with malaria have higher H3K4me3, an epigenetic modification to the nuclear histone H3, compared to healthy North American adult controls [13]. Epigenetic reprogramming underlies the induction of trained immunity [2]. These epigenetic changes result in unfolding of chromatin regions that enhances transcription and gene expression of factors involved in the immune response. The changes are only partially removed after the primary stimulus is eliminated. Nonetheless, the remaining changes may be enough to result in a strong, rapid immune response of the stimulated cells to challenge secondary stimuli (e.g., SARS-CoV-2) [1].

Studying the seasonal variation in the incidence of malaria and COVID-19 could have provided a better picture of the situation. If the hypothesis presented is correct and malaria could prevent SARS-CoV-2 infection, it is expected that the incidence of COVID-19 would decrease soon after the rain or during the period with high incidence of malaria. That might be considered a limitation of this work, but based on the hypothesis, stimulation of the innate immunity results in a trained immunity that would last for a period, at least 6 months for OPV [4]. This sustained immunity would abolish the changes, if any, in the incidence of COVID-19—a single exposure to malaria could be enough to cause the trained immunity that would last for a couple of months. This would be more pronounced in regions endemic for malaria where people are constantly exposed to the parasite. Another limitation of the study would be the nature of the study design. No causal inference could be made from an ecological study. The design is just hypothesis-generating. The coverage of vaccination against COVID-19 was not also considered in our study. However, at the time of our data collection (April 19, 2021), most African countries either did not introduce COVID-19 vaccines or immunized a very small part of their populations.

## Conclusions

African countries with higher prevalence of malaria had a lower incidence of COVID-19. Malaria might protect people against SARS-CoV-2 through the stimulation of innate immunity. Stimulation of the innate immune system can be done in various ways. This could be the first line of defense in the pandemic preparedness arsenal.

## Supplementary Information

**Additional file 1: Figure S1.** QQ-plot of the eight studied continuous variables. Cases/100,000 is cumulative incidence of COVID-19 per 100,000 population and HDI, the human development index. **Figure S2.** Distribution of data points as well as the box and whisker plot indicating the cumulative incidence of COVID-19 per 100,000 population in countries with malaria prevalence < 3% and ≥ 3% (arbitrary chosen cut-off value). The horizontal line in the middle of each box indicates the median; the bottom border, the 25th percentile; the top border, 75th percentile; the lower whisker, the smallest data point within 1.5 × the interquartile range (IQR) less than the 25th percentile; and the upper whisker, the largest point within 1.5 × IQR greater than the 75th percentile. Points smaller than the lower whisker and greater than the upper whisker were considered outliers; all outliers were included in data analyses. *HDI: human development index. Data Dictionary. Negative binomial regression model used in the current study using the original dataset.

**Additional file 2:** Raw data for the studied African countries.

## Data Availability

Parts of the codes developed in *R* language, are provided in the Additional file 2. The raw dataset compiled from publicly available data sources (“Our World in Data” website, available from https://ourworldindata.org/covid-cases and https://ourworldindata.org/grapher/covid-stringency-index; Global Burden of Disease (GBD 2019) Collaborative Network website, available from https://vizhub.healthdata.org/gbd-results/; and the Natural Earth, available from https://www.naturalearthdata.com/) are also available as Additional file 2. Complete *R* codes are available from the corresponding author, upon reasonable request.

## Abbreviations

BCG: Bacillus Calmette-Guérin
MMR: Measles, mumps, and rubella
OPV: Oral polio vaccine
SARS-CoV-2: Severe acute respiratory syndrome coronavirus 2
COVID-19: Coronavirus disease 2019
GDP: Gross domestic product
HDI: Human development index
GPEI: World Health Organization Global Polio Eradication Initiative
IQR: Interquartile range
CI: Confidence interval
UNDP: United Nations Development Programme
