# Supplementary material for: Malaria and the incidence of COVID-19 in Africa: an ecological study
Source: BMC Infect Dis. 2023 Feb 3;23:66. doi: 10.1186/s12879-023-08032-2 (PMC9896446; doi:10.1186/s12879-023-08032-2)
Supplement: Supplementary file 1 — Additional file 1: Figure S1. QQ-plot of the eight studied continuous variables. Cases/100,000 is cumulative incidence of COVID-19 per 100,000 population and HDI, the human development index. Figure S2. Distribution of data points as well as the box and whisker plot indicating the cumulative incidence of COVID-19 per 100,000 population in countries with malaria prevalence < 3% and ≥ 3% (arbitrary chosen cut-off value). The horizontal line in the middle of each box indicates the median; the bottom border, the 25th percentile; the top border, 75th percentile; the lower whisker, the smallest data point within 1.5 × the interquartile range (IQR) less than the 25th percentile; and the upper whisker, the largest point within 1.5 × IQR greater than the 75th percentile. Points smaller than the lower whisker and greater than the upper whisker were considered outliers; all outliers were included in data analyses. *HDI: human development index. Data Dictionary. Negative binomial regression model used in the current study using the original dataset. [file 12879_2023_8032_MOESM1_ESM.docx]

Additional file 1


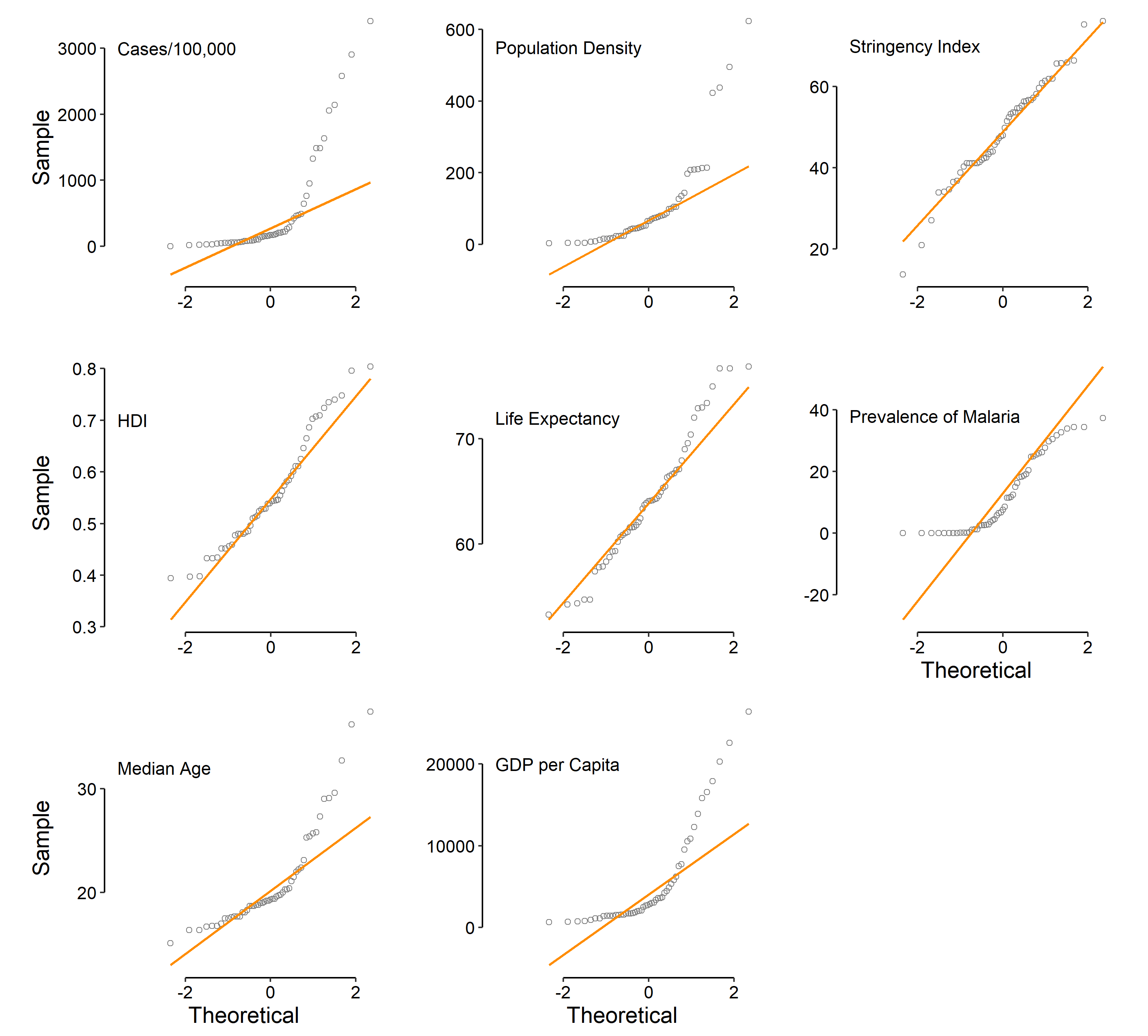


**Figure S1.** QQ-plot of the eight studied continuous variables. Cases/100,000 is cumulative incidence of COVID-19 per 100,000 population and HDI, the human development index.


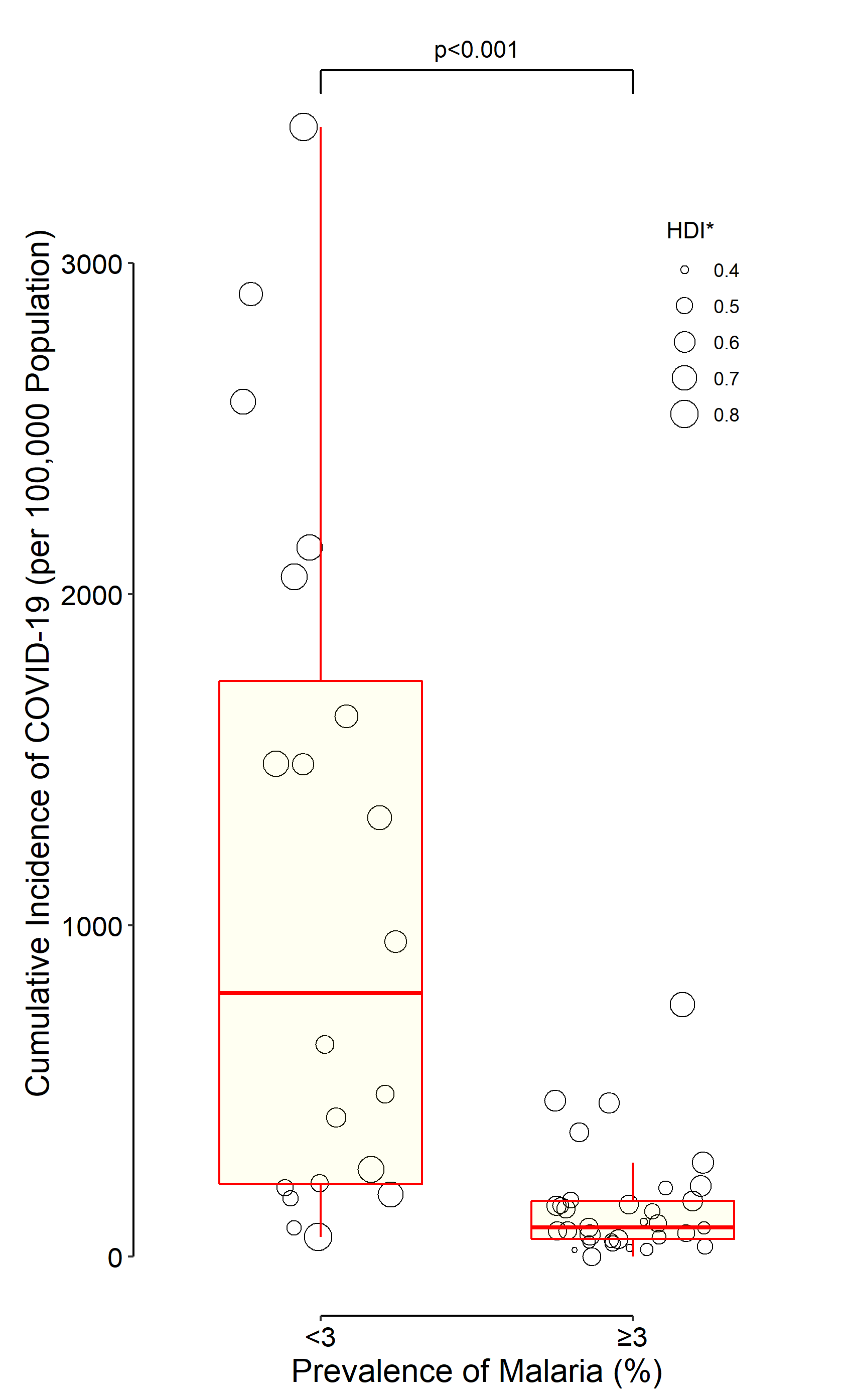


**Figure S2.** Distribution of data points as well as the box and whisker plot indicating the cumulative incidence of COVID-19 per 100,000 population in countries with malaria prevalence < 3% and ≥ 3% (arbitrary chosen cut-off value). The horizontal line in the middle of each box indicates the median; the bottom border, the 25^th^ percentile; the top border, 75^th^ percentile; the lower whisker, the smallest data point within 1.5 × the interquartile range (IQR) less than the 25^th^ percentile; and the upper whisker, the largest point within 1.5 × IQR greater than the 75^th^ percentile. Points smaller than the lower whisker and greater than the upper whisker were considered outliers; all outliers were included in data analyses. *HDI: human development index.

**Data Dictionary**

HDI Human development index

CasesPer100 Cumulative incidence of COVID-19 per 100,000 population

PrevMal Prevalence of malaria

**Negative binomial regression model used in the current study using the original dataset:**

*fit <- glm.nb(CasesPer100 ~ PrevMal + HDI, data = dat)*

*summary(fit)*

Call:

glm.nb(formula = CasesPer100 ~ PrevMal + HDI, data = dat, init.theta = 1.525947044, link = log)

Deviance Residuals:

Min 1Q Median 3Q Max

-3.5849 -0.8323 -0.1365 0.3440 1.5414

Coefficients:

Estimate Std. Error z value Pr(>|z|)

(Intercept) 1.14133 0.79045 1.444 0.1488

PrevMal -0.03227 0.01084 -2.978 0.0029 **

HDI 8.73158 1.25278 6.970 3.17e-12 ***

---

Signif. codes: 0 ‘***’ 0.001 ‘**’ 0.01 ‘*’ 0.05 ‘.’ 0.1 ‘ ’ 1

(Dispersion parameter for Negative Binomial(1.5259) family taken to be 1)

Null deviance: 159.698 on 51 degrees of freedom

Residual deviance: 58.016 on 49 degrees of freedom

(1 observation deleted due to missingness)

AIC: 693.14

Number of Fisher Scoring iterations: 1

Theta: 1.526

Std. Err.: 0.280

2 x log-likelihood: -685.140
